# The COVID-19 pandemic could be a setback for gender equality in oncology—and a key moment to push ahead with transformative policies

**DOI:** 10.1016/j.esmoop.2021.100169

**Published:** 2021-06-02

**Authors:** A. Cervantes

**Affiliations:** Hospital Clinic Universitario, Biomedical Research institute INCLIVA, University of Valencia, Valencia, Spain

The indirect effects on global healthcare—and on the medical oncology community—of the COVID-19 pandemic and national lockdowns enforced to curb its spread are only just beginning to be understood. The reorganisation of medical services has often meant increased workloads and difficult working conditions for practitioners in the clinic, while the shutdown of schools, childcare facilities and certain non-urgent health services has also left many people with additional caretaking duties to fulfil at home. As European Society for Medical Oncology (ESMO) President-Elect, and as a person who takes care of others for a living, I am troubled by the results of a survey conducted by the ESMO Women for Oncology (W4O) Committee about the impact of the outbreak on the lives of oncologists, which confirm what some had feared: that the burden has fallen disproportionately on our female colleagues, with concerning implications for their future career trajectories and gender equality in our profession.

Almost three-quarters of the 649 respondents to the survey, available online in June of last year, were women. Unfortunately, this is consistent with the low male participation rates observed previously in the W4O Committee's activities, such as the discussions of the W4O Forum at the annual ESMO Congress. It highlights once again that men are not sufficiently engaged in gender issues, even though these are consequential for the entire oncology community.

The authors suggest, however, the imbalance in participation could also indicate that women were more motivated to respond insofar as they suffered more than men from the fallout of the pandemic. While the majority of those surveyed reported being adversely affected in their family, personal and professional lives, the proportion of women stating this was significantly bigger than the share of men in all three areas. Women were more likely than their male counterparts to have spent less time on self-care both during and after the period of confinement, but they also more frequently worked longer hours on hospital and laboratory tasks. Meanwhile, more than one-third of women in the study dedicated less time to science during the health crisis compared with only one-quarter of men, confirming initial assessments that had alerted us to a drop in female authorship in scientific publications during the first peak of the outbreak.^1^

Although the shift in priorities at the height of the emergency understandably impacted research productivity across the board, the gender discrepancy observed here appears to have persisted past the lockdowns and into the period of loosened restrictions: the damage may even be permanent for those in the early or critical stages of their career. This insight should be alarming enough to warrant further research into the factors that lead to these disparities, because we cannot afford to lose talented women who may drive development and advances in all aspects of our profession. ESMO has published a number of articles on the activities of the W4O Committee, which is actively investigating the challenges faced by female oncologists,^2^ and it will be important to continue this work in the future.

One thing we do know is that gender transformative policies can be effective for keeping high-potential individuals involved and motivated in oncology. ESMO has taken action in this area, too: the ESMO Women for Oncology Award is presented every year at the ESMO Congress to an individual who has contributed to supporting the career development of women working in oncology. The W4O Committee is dedicated to empowering female oncologists everywhere, for example by offering guidance on setting up and running local women's networks and initiatives through its W4O Hub, a community where women working in oncology can find support to translate universal issues into national or regional discussions and solutions. Its mentorship sessions at ESMO events also allow young doctors to meet inspirational female leaders and have their questions answered by more experienced women within the oncology community. And because true equality can only be attained through diversity, W4O aims to include more and more men in the conversation: five male colleagues have joined the Committee, which strives to publish inclusive content that is meaningful to all oncologists.

The Society is also committed to enabling more female talent to rise through the ranks by promoting equal access to career development programmes like ESMO's leadership training—the ESMO Leaders Generation Programme—and to fostering inclusiveness throughout its own organisation by bringing men and women from around the world into its Committees and Faculty. At the highest level of our Society, the ESMO Executive Board has achieved gender parity, reflecting the evolution of our membership from just 20% of women in the year 2000 to almost half of the ESMO community being female today.^3^ The importance of this milestone should not be underestimated, as men continue to outnumber women on the boards of most organisations—and most oncology societies—in Europe and around the world.

However, there is still much to be done and this new data should serve as a stark reminder that progress is not irreversible—that we must double down to ensure that what has been acquired is not lost and continue to work towards more diversity across our profession. At national and international oncology meetings, for instance, the proportion of female speakers is still too often lower than in the community at large. Women accounted for only 26% of speaking engagements at the ESMO Congress 2016^4^ and while we have seen that proportion increase at subsequent events, progress has been frustratingly slow. That is why the Society is actively seeking to attract more talented women to the scientific committees, to positions as moderators and lecturers at our meetings all over the world.

The pandemic has been an acutely stressful situation, but as oncologists we will encounter many other challenges in our professional lives. This career is a long and committed one, involving continuous exposure to patients at difficult moments in their lives, some of whom will experience poor outcomes. Going forward, we cannot tolerate that this crisis or those to come exact a heavier toll from our female colleagues. As President-Elect of a Society in which women now make up the majority of members under the age of 40 years, I am conscious of my responsibility to ensure that ESMO adequately represents and supports them in years to come.
